# Caseous Calcification: Now You See Me, Now You Don’t

**DOI:** 10.7759/cureus.20911

**Published:** 2022-01-03

**Authors:** Joel Thekekara, Jack Xu, Chris Baker, Angel López-Candales

**Affiliations:** 1 Department of Internal Medicine, Cleveland Clinic, Cleveland, USA; 2 Department of Internal Medicine, University of Arkansas for Medical Sciences, Little Rock, USA; 3 Cardiac Noninvasive Laboratory, University of Arkansas for Medical Sciences, Little Rock, USA; 4 Cardiovascular Medicine, University of Missouri Kansas City, Kansas City, USA

**Keywords:** caseous calcification, echocardiography, valvular mass, mitral annular calcification, mitral annulus

## Abstract

Caseous calcification of mitral annulus is a rare variant of mitral annulus calcification that can mimic infective endocarditis, myocardial abscess, valve myxoma, or papillary fibroelastoma. On transthoracic echocardiography, the mass appears as a large, round echodense structure with a large calcification and central echolucency.

We present a case of a 72-year-old female with a past medical history significant for diabetes mellitus, hypertension, and end-stage renal disease who was noted to have caseous calcification of the mitral annulus on transthoracic echocardiography, which was done as part of a preoperative kidney transplantation evaluation. The mass spontaneously resolved before the planned mitral valve surgery.

Caseous calcification of mitral annulus should be considered in the differential for a cardiac mass, particularly if it is attached to the posterior aspect of the mitral valve. Accurate identification of this rare cardiac mass is essential to avoid unnecessary surgical intervention as clinical course is usually benign.

## Introduction

Involvement of the fibrous support of the mitral valve most commonly involves the posterior portion of the annulus as a result of mitral annular calcification (MAC), which is known to be a chronic, degenerative process [1]. In contrast, caseous mitral annular calcification (CMAC), also known as liquefaction necrosis of MAC, is by far an uncommon entity [2].

While MAC is usually visualized as an irregular mass density mainly involving the posterior aspect of the mitral valve annulus, it is also associated with acoustic shadowing [3]; CMAC has been described as large echogenic mass with a central echo-lucent area without acoustic shadowing [4].

Even when CMAC is generally considered a rare echocardiographic finding, its size, mobility, and location can increase the incidence of stroke and mitral regurgitation [5]. Aside from the potential clinical complications, the most important issue is to arrive at the correct diagnosis while avoiding mistaking CMAC for a tumor, abscess, vegetation, or thrombus [6].

In this case report, we present the case of a patient in whom a large mobile, dense mass was incidentally identified on a routine echocardiogram obtained as part of preoperative evaluation for possible kidney transplantation.

## Case presentation

We present the case of a 72-year-old female with a past medical history significant for diabetes mellitus, hypertension, and end-stage renal disease (ESRD), not on dialysis, who was initially seen for pre-kidney transplant evaluation.

**Figure 1:**
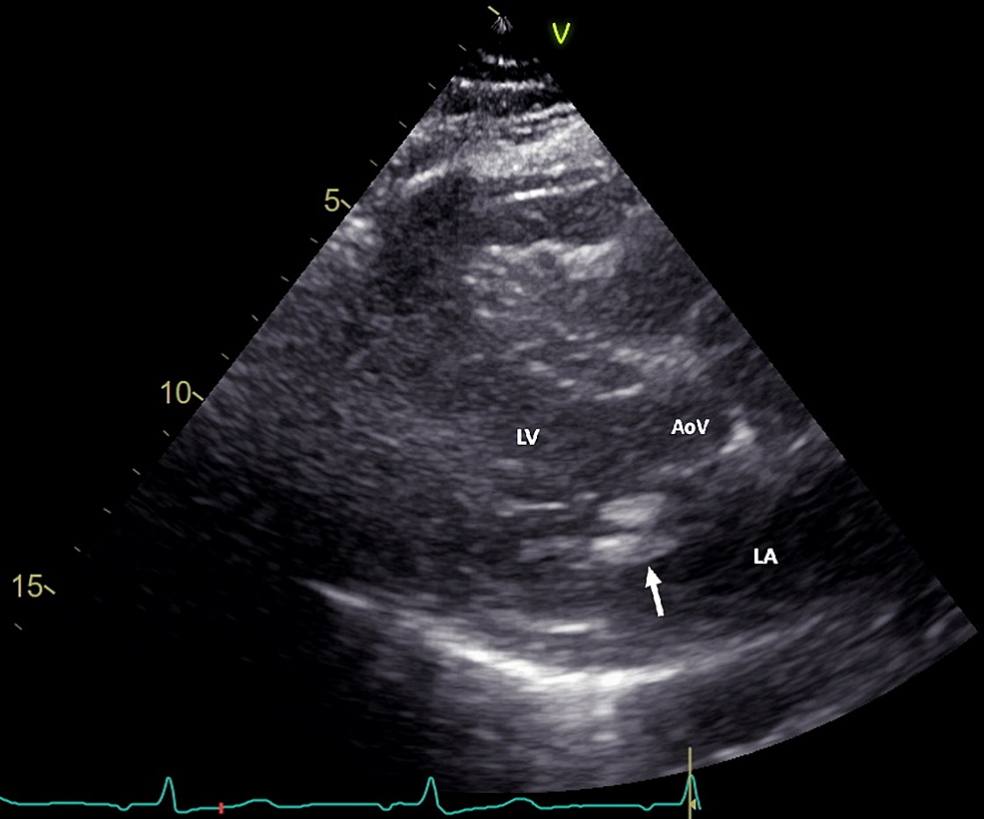
Transthoracic echocardiogram showing a parasternal long-axis view image of the heart. The white arrow shows the large calcified mass attached to the posterior aspect of the mitral valve. LA = left atrium, LV= left ventricle, AoV = aortic valve.

An echocardiogram, although technically challenging, showed a 1.0 cm by 0.7 cm, regularly shaped homogeneous, echo-bright sessile mass attached to the posterior aspect of the mitral annulus as seen in Figure 1. In addition, color flow showed only a trace amount of mitral regurgitation. Left atrial size was normal so as the right ventricular systolic pressure. The rest of the echocardiographic examination revealed a trileaflet aortic valve that was simply mildly thickened without any significant calcification. Both tricuspid and pulmonary valves were normal. No other masses were identified. Finally, normal left ventricular cavity dimensions with mildly increased left ventricular wall thickness and overall normal left ventricular systolic function were noted.

**Figure 2:**
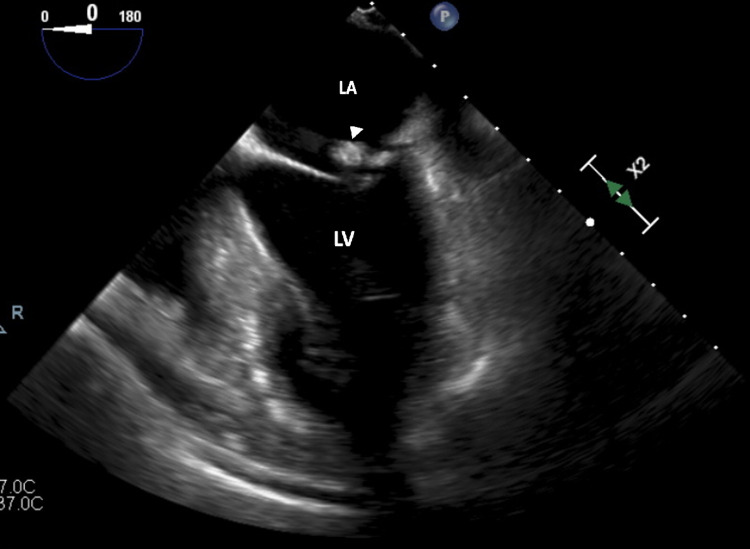
Transesophageal mid-esophageal view at zero-degree orientation showing the annular density CMAC (arrowhead). Though the orientation has changed, chamber labeling remains the same. LA = left atrium, LV= left ventricle, CMAC = caseous mitral annular calcification.

**Figure 3:**
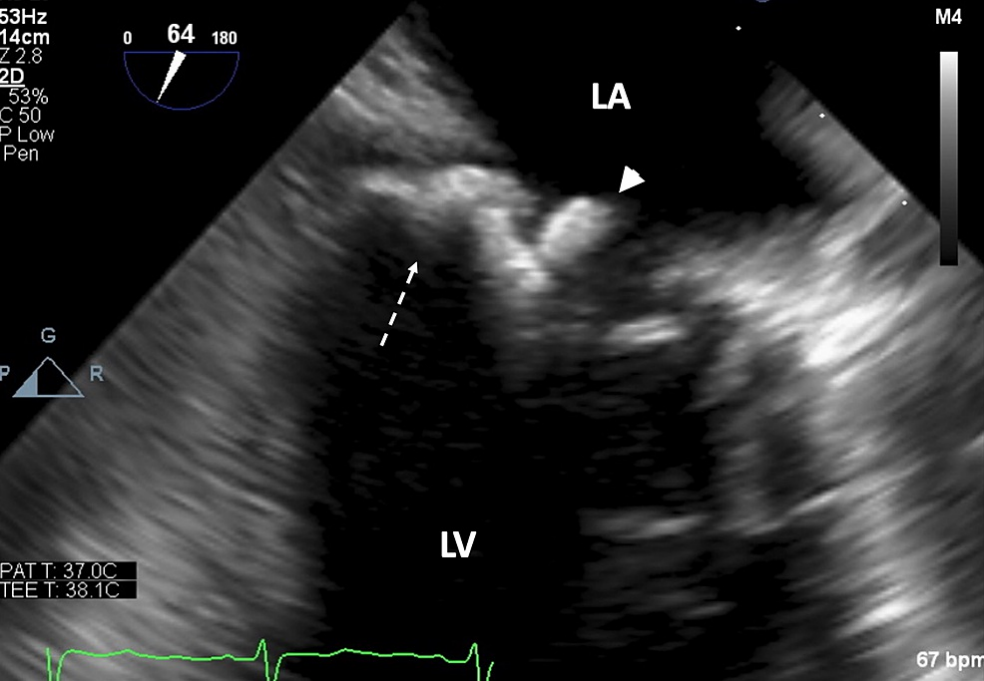
Transesophageal mid-esophageal view at 64 degree orientation showing both the MAC (broken arrow) and the caseous mass (arrowhead). LA = left atrium, LV= left ventricle, MAC = mitral annular calcification.

**Figure 4:**
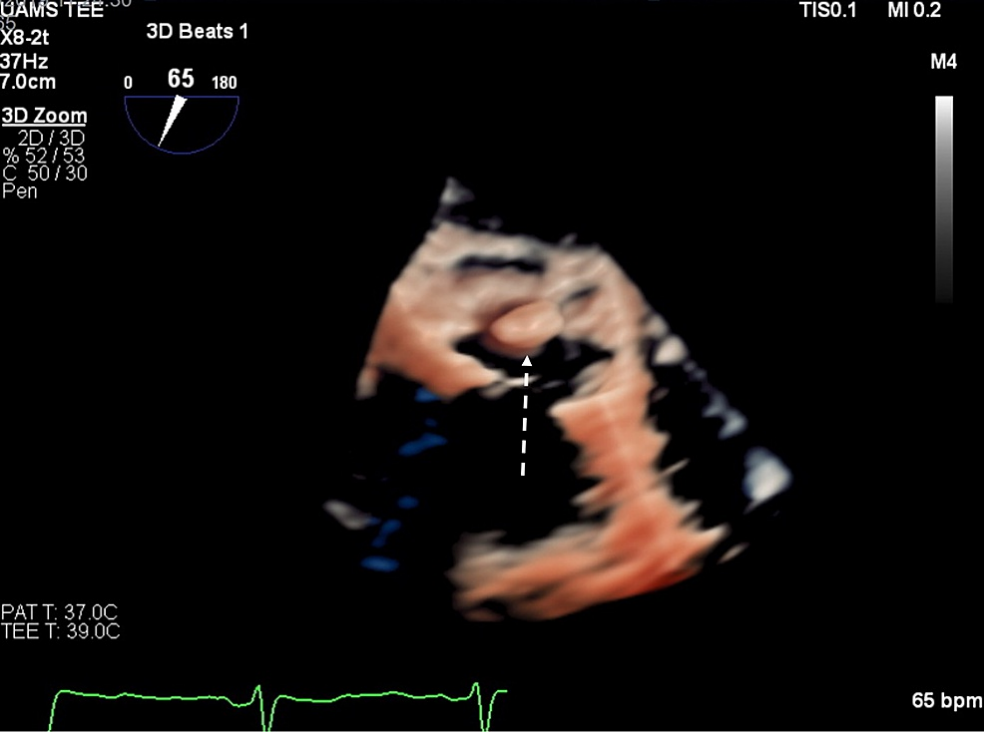
Three-dimensional view of the CMAC attached to the posterior aspect of the mitral annulus (arrowhead). CMAC = caseous mitral annular calcification.

This unexpected finding prompted the following differential diagnosis: large vegetation, thrombus, valve myxoma, atypical fibroelastoma, or simply an unusually large calcified mass. Upon review of prior studies this was not present on a previous study obtained three years prior. A transesophageal echocardiogram (TEE) was done, which verified the presence of the mass (Figures 2-4).

**Figure 5:**
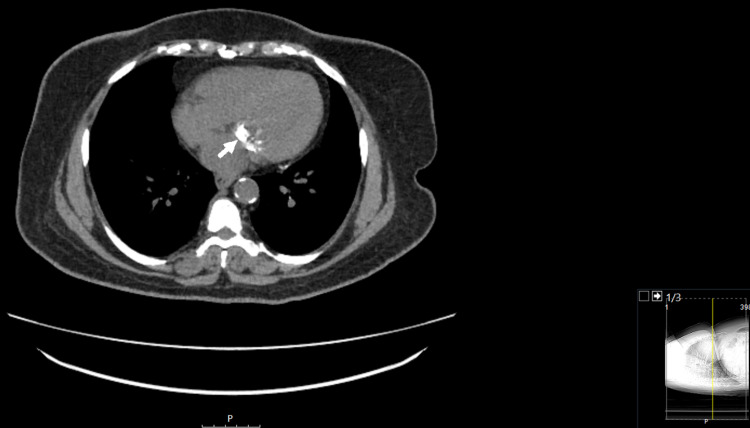
A computed tomographic chest scan view without contrast. 1 mm slices taken showing dense mitral valve annular calcification predominantly involving the inferior aspect of the annulus (white arrow). Calcifications in the aorta are also seen.

A chest computed tomography scan better characterized this finding as a dense MAC predominantly involving the inferior aspect (Figure 5). Moderate calcification of the coronary arteries and the aorta was also seen.

**Figure 6:**
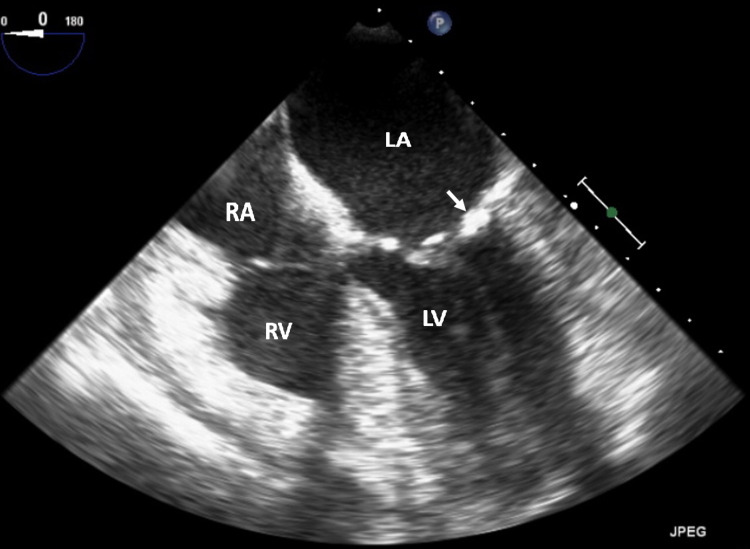
Transesophageal mid-esophageal view at zero-degree orientation showing MAC (white arrow) without the previously seen CMAC density. RA = right atrium, LA = left atrium, LV = left ventricle, RV = right ventricle, MAC = mitral annular calcification, CMAC = caseous mitral annular calcification.

**Figure 7:**
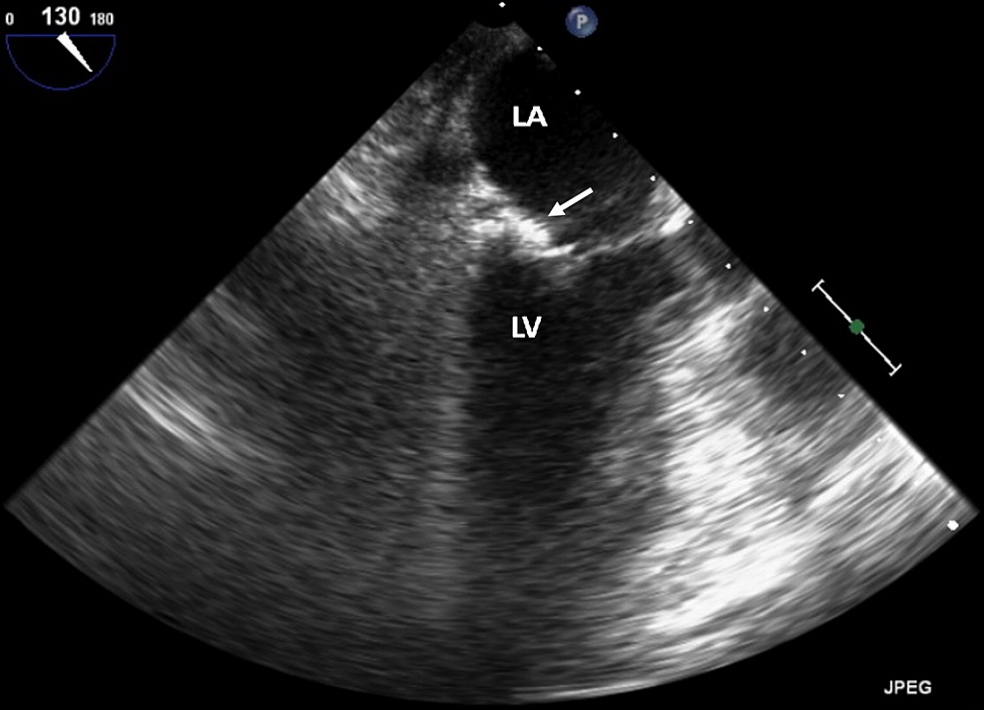
Transesophageal mid-esophageal view at 130 degree orientation with preferential view of the posterior aspect of the mitral valve simply showing showing MAC (white arrow). LA = left atrium, LV = left ventricle, MAC = mitral annular calcification.

**Figure 8:**
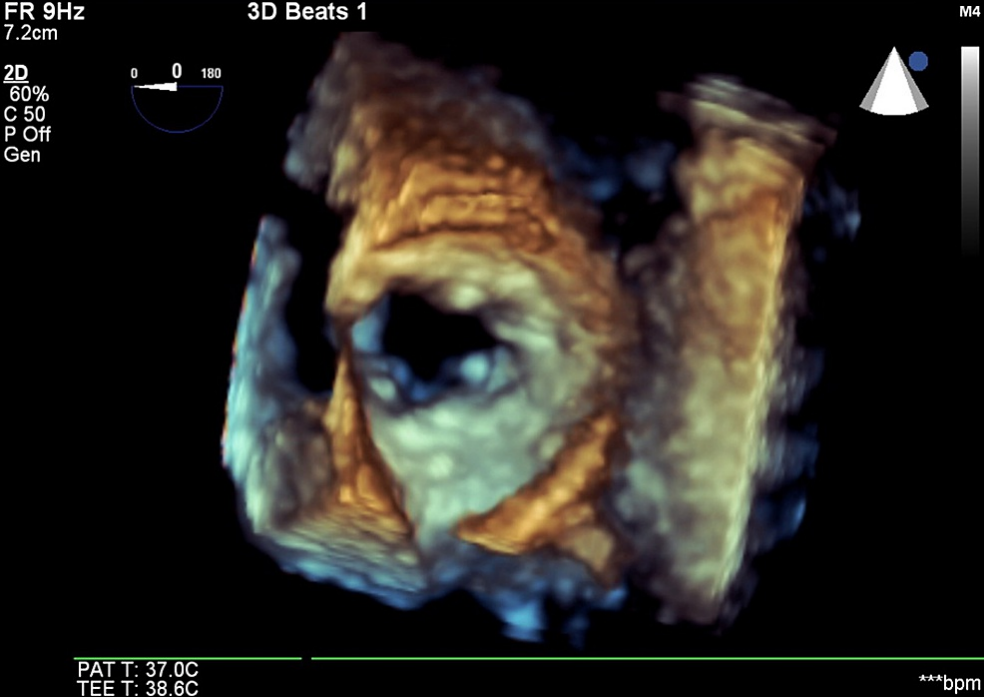
Three-dimensional view of the mitral annulus, from the surgeon’s view. Please note the calcification but no additional mass seen this time.

She underwent a coronary angiogram that showed multivessel disease with a moderate long lesion in the distal left anterior descending artery and severe lesions in the circumflex and right posterior descending arteries. Based on her coronary artery disease (CAD), she was referred for coronary artery bypass surgery (CABG) for treatment for her CAD. Then based on all the imaging findings, it was suggested to the cardiac surgeon to revise the mitral valve at the time of the surgery to consider the best approach not only depending on tissue samples that would have been taken at the time of the exploration but also on the valve anatomy. However, on the day of surgery (exactly 46 days after her initial transthoracic echocardiogram [TTE]), she underwent a repeat TEE as customarily obtained, which showed spontaneous resolution of the previously identified mass (Figures 6-8). The decision was made at that time, to cancel her CABG and mitral valve revision based on the unquestionable TEE and 3D findings and her CAD was later treated with multivessel percutaneous coronary interventions.

## Discussion

MAC is a chronic degenerative process usually localized to the posterior portion of the mitral valve fibrous ring [6]. In contrast, CMAC is a lesser-known and rare variant of MAC, seen in 0.64% of all MACs [6,7]. The prevalence of CMAC is higher in the elderly and in ESRD patients [6].

The most common presentation of CMAC is by an incidental finding on a TTE [6]. CMAC appears as a large, round echodense mass with calcifications in the borders and central echolucency without an acoustic shadow [6-10]. The central echolucency is representative of liquefaction [6,7]. In contrast, MAC appears as an echodense structure with an irregular, lumpy appearance with an associated acoustic shadow [7].

CMAC can be confused with infective endocarditis, myocardial abscess, valve myxoma, or papillary fibroelastoma [6]. It is important to differentiate CMAC from these other etiologies as management can be vastly different. Clinical presentation is vital in arriving at the accurate diagnosis. Infective endocarditis can present as an oscillating mass on a support structure, but is usually associated with fever, positive blood cultures, and valvular regurgitation [6,7]. Myocardial abscess appears as a homogeneous echogenic mass within the myocardium or annular region [6]. It can be differentiated from CMAC by the presence of systolic blood flow via color Doppler and lack of calcification [6]. Myxoma commonly arises from the left atrium, but a myxoma arising from the mitral valve has rarely been reported [8]. Valvular myxoma appears as a heterogeneous larger mass with patchy echolucent areas [8]. It can be differentiated from CMAC by lack of calcification in the borders. Papillary fibroelastoma can be differentiated from valvular myxoma and CMAC by the presence of homogeneous, speckled, pedunculated mobile mass on echocardiography [8].

In our case, CMAC spontaneously resolved before her planned surgery, which is a rare phenomenon that has been described before in three separate case reports [7,9,10]. In cases described by Poh et al. and Correale et al. [7,9], spontaneous resolution was noted several months later with conservative treatment. In both cases, the mechanism of resolution was hypothesized to be central liquefaction and dissolution of the material through a rupture of exterior wall without clinical consequences. Koito et al. [10] described a case of CMAC that resolved after treatment with hemodialysis with low calcium concentration. In our case, there was no change in management after the incidental finding of the CMAC on preoperative echocardiography. So, we believe that spontaneous resolution of the central area of liquefaction with complete dissolution of the entire caseous material is the most likely mechanism explaining the disappearance of the mitral annular mass prior to surgery.

## Conclusions

In conclusion, CMAC is a rare form of MAC that is most likely found as an incidental finding on routine echocardiography. This valvular mass can easily be confused with other lesions such as valvular vegetations, myocardial abscess, valve myxoma, or papillary fibroelastoma. Accurate identification is essential to avoid unnecessary interventions. Although the clinical course for CMAC is probably underappreciated as a source of potentially serious complications, for the most part, it is benign with spontaneous resolution, as seen in our patient.

Further research is needed to elucidate the mechanisms of progression from MAC to CMAC. The relationship between serum calcium levels and CMAC formation is another aspect that needs to be further investigated.
